# Supplementary material for: Caffeic Acid-Zinc Basic Salt/Chitosan Nanohybrid Possesses Controlled Release Properties and Exhibits In Vivo Anti-Inflammatory Activities
Source: Molecules. 2023 Jun 24;28(13):4973. doi: 10.3390/molecules28134973 (PMC10343822; doi:10.3390/molecules28134973)
Supplement: Supplementary file 1 [file molecules-28-04973-s001.zip › molecules-2410244-supplementary.pdf]

Supplementary Materials

Caffeic Acid-Zinc Basic Salt/Chitosan Nanohybrid Possesses Controlled Release Properties and Exhibits In Vivo Anti-inflammatory Activities

Carla Carolina Ferreira Meneses, Paulo Robson Monteiro de Sousa, Kely Campos Navegantes-Lima, Lisa Maria Mendes de Almeida Souza, Waldeci Paraguassu Feio, Claudio Marcio Rocha Remedios, Jenny Jouind, Philippe Thomasd, Olivier Massond, Cláudio Nahum Alvesa, Jerônimo Lameira, Marta Chagas Monteiro

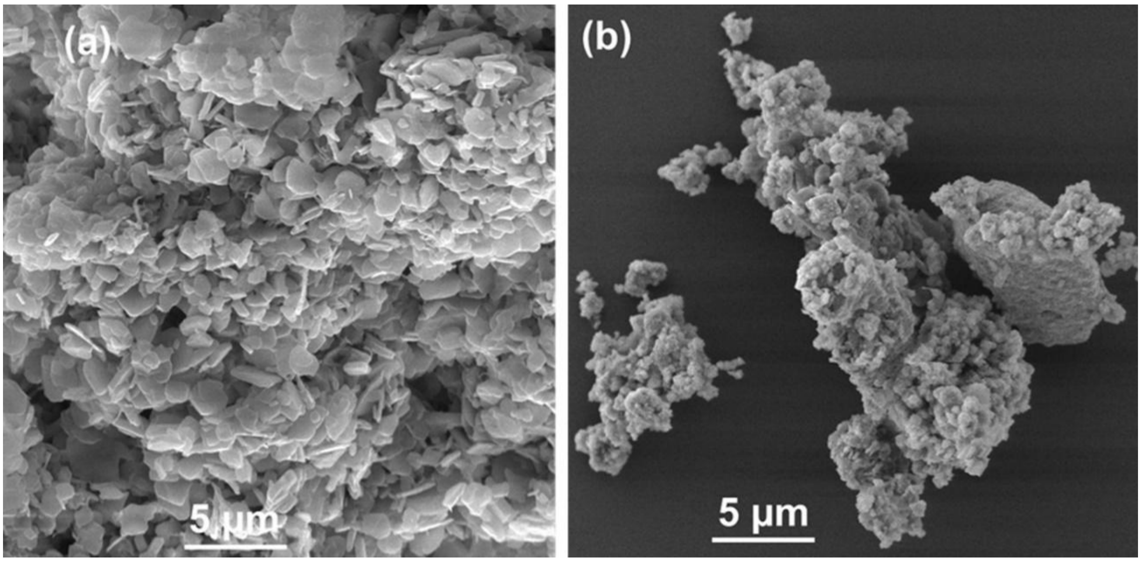

Figure S1. Scanning electron micrographs of (a) pristine ZBS and (b) ZBS-CA sample.
